# Control of Intracellular Calcium Signaling as a Neuroprotective Strategy

**DOI:** 10.3390/molecules15031168

**Published:** 2010-03-03

**Authors:** R. Scott Duncan, Daryl L. Goad, Michael A. Grillo, Simon Kaja, Andrew J. Payne, Peter Koulen

**Affiliations:** Vision Research Center and Departments of Ophthalmology and Basic Medical Science, School of Medicine, University of Missouri, 2411 Holmes Street, Kansas City, MO 64108, USA; E-Mails: duncanrs@umkc.edu (R.S.D); goadd@umkc.edu (D.L.G); mag6y5@umkc.edu (M.A.G.); kajas@umkc.edu (S.K.); paynea@umkc.edu (A.J.P.)

**Keywords:** calcium, Ca^2+^, intracellular calcium channel, ion channel, extracellular, intracellular, accessory proteins, associated proteins, G-protein coupled receptors, imaging, microscopy, signaling, neuroprotection, cytoprotection, neurodegeneration, Alzheimer's disease, Huntington’s disease, retina

## Abstract

Both acute and chronic degenerative diseases of the nervous system reduce the viability and function of neurons through changes in intracellular calcium signaling. In particular, pathological increases in the intracellular calcium concentration promote such pathogenesis. Disease involvement of numerous regulators of intracellular calcium signaling located on the plasma membrane and intracellular organelles has been documented. Diverse groups of chemical compounds targeting ion channels, G-protein coupled receptors, pumps and enzymes have been identified as potential neuroprotectants. The present review summarizes the discovery, mechanisms and biological activity of neuroprotective molecules targeting proteins that control intracellular calcium signaling to preserve or restore structure and function of the nervous system. Disease relevance, clinical applications and new technologies for the identification of such molecules are being discussed.

## 1. Introduction

Changes in the intracellular concentration of Ca^2+^ control a plethora of cellular and physiological processes, including neurotransmitter release, hormone secretion, cell fate and gene expression. As such, the control of the intracellular Ca^2+^ concentration is critical to the functioning and survival of the cell. In both acute and chronic degenerative diseases of the nervous system the viability and function of neurons is reduced as a result of changes in intracellular calcium signaling. In particular, pathological increases in the intracellular calcium concentration promote such pathogenesis. Furthermore, numerous regulators of intracellular calcium signaling located on the plasma membrane and intracellular organelles have been identified to be involved in many of these pathophysiological processes. Neuroprotective strategies targeting different aspects of the complex control of intracellular Ca^2+^ signaling are a promising approach for pharmaceutical intervention in degenerative diseases of the nervous system. To this end, diverse groups of chemical compounds targeting ion channels, G-protein coupled receptors, pumps and enzymes have been identified as potential neuroprotectants, each targeting different aspects of fine-tuned control of the intracellular Ca^2+^ concentration. The present review summarizes the discovery, mechanisms and biological activity of representative neuroprotective molecules targeting proteins that control intracellular calcium signaling to preserve or restore structure and function of the nervous system. Furthermore, disease relevance, clinical applications and new technologies for the identification of such molecules are being discussed.

## 2. Intracellular Calcium Channels as Targets for the Development of Neuroprotective Strategies

Inositol 1,4,5-trisphosphate (IP_3_) receptors (IP_3_Rs) and ryanodine receptors (RyRs) are the two major intracellular Ca^2+^ channels (ICCs) that release Ca^2+^ from intracellular stores such as the endoplasmic reticulum (ER). Release and sequestering of Ca^2+^ from these intracellular stores is a major determinant in the shape and resting state of intracellular Ca^2+^ levels. IP_3_Rs and RyRs are critical for Ca^2+^ dependent processes such as cellular growth, development, gene expression and neurotransmission [1,2,3,4,5,6,7,8,9,10]. Aberrant ICC function as a controlling part of overall intracellular Ca^+2^ handling also plays a role in a variety of neurodegenerative diseases including Alzheimer’s disease (AD), Huntington’s disease (HD), Amyotrophic lateral sclerosis (ALS), excitotoxicity induced apoptosis, hypoxia, and neuroinflammation [11,12,13,14,15,16,17,18]. 

The role of ICCs in AD pathology has become more apparent in the last several years. For example, phosphatidylinositol and IP_3_ levels are reduced in the brains of patients with AD. The decrease in IP_3_ binding sites in brains of AD patients correlates with the increased occurrence of amyloid plaques and neurofibrillary tangles [19,20,21,22,23,24]. In addition, IP_3_R activity is altered in cultured neurons from mouse models of AD [13,16,17] and in cortical neurons exposed to β-amyloid protein (Aβ) resulting in increased neuronal death [16]. Mutations in presenilin-1 (PS-1), presenilin-2 (PS-2), or amyloid precursor protein (APP) lead to early adult onset AD, characterized by Aβ plaques, neuroinflammation, and increased oxidative stress. These mutations have also been linked to alterations in intracellular Ca^2+^ homeostasis [25], with PS1 and PS2 mutations showing alterations in IP_3_R activity resulting in enhanced Ca^2+^ release from ER stores [31,32].

In addition, RyR function is directly affected by wild type PS1 [27] and PS2 [28] with mutations in these two proteins leading to enhanced Ca^2+^ release [29] and vulnerability of cells to apoptosis [30]. Transgenic mutant APP, PS-1, or triple transgenic (PS-1/PS-2/APP) mice exhibit elevated RyR expression, RyR-mediated Ca^2+^ release, and susceptibility to Aβ [14,26]. This is supported clinically, as AD patients displaying advanced cognitive decline exhibit changes in neuronal RyR expression and ryanodine binding in specific brain regions where Aβ deposition occurs [11]. Aβ treatment of cultured neurons increases RyR3 expression and RyR-mediated Ca^2+^ release contributing to neuronal cell death [16,18].

HD is a neurodegenerative disorder characterized by the loss of striatal neurons, neuro-inflammation, and increased oxidative stress [33,34]. HD is caused by a polyglutamine expansion of the amino terminus of the Huntington protein (Htt), which promotes binding to Huntington-associated protein (HAP1) [35]. In a yeast two-hybrid screen the C-terminus of brain IP_3_R type 1 (IP_3_R1) associates with the HD Htt-HAP1 complex and increases IP_3_R1 sensitivity to activation by IP_3 _[36]. This sensitization of the IP_3_R leads to elevated Ca^2+^ release and apoptosis in neuronal cells [15]. Furthermore, studies with the YAC128 transgenic HD mouse model have shown that disruption of IP_3_R binding to Htt reduces glutamate induced apoptosis by stabilizing intracellular Ca^2+^ homeostasis [37]. Genomic analysis of the R6/2 mouse HD model shows a downregulation in RyR type 1 expression further implicating intracellular Ca^2+^ induced Ca^2+^ release (CICR) in the progression of this disease [38].

Amyotrophic lateral sclerosis (ALS) is defined by loss of motor neurons, neuroinflammation, and increased oxidative stress. T cell dysfunction and mutations in the superoxide dismutase 1 (SOD-1) gene have been suggested as possible mechanisms for the progression of this disorder [34,39]. Abnormal IgG from sporadic ALS patients interacts with Cav2.2 N-type calcium channels in motor neurons causing an increase in intracellular Ca^2+^. This increase in Ca^2+ ^can be ablated by the use of ryanodine and IP_3_ receptor blockers, suggesting a role for altered CICR and intracellular Ca^2+ ^signaling in ALS [40].

These studies provide a rationale for determining the roles of ICCs in the progression of neurodegenerative diseases as well as for the development of neuroprotective strategies and potential therapeutic agents with ICCs and their signaling and binding partners as targets.

## 3. VGCCs as Targets for the Development of Neuroprotective Strategies

Increase of the intracellular Ca^2+^ concentration is widely considered the single most important contributor to neurodegeneration and neuronal cell death following ischemic or hypoxic insult [237]. As a consequence, voltage-gated Ca^2+^ channels (VGCCs) are an important target for the development of neuroprotective strategies, given their central role in depolarization-induced Ca^2+^ influx [41]. 

VGCCs comprise two groups of high voltage-activated channels, dihydropyridine-sensitive Cav1 (L-type) channels and Cav2 (P/Q-, N- and R-type) channels, as well as low voltage-activated Cav3 (T-type) channels. In addition to the pore-forming α_1_-subunit, high voltage-activated Ca^2+^ channels form a complex consisting of an additional β-, α_2_δ- and possibly γ-subunit. The biophysical properties of VGCCs are largely determined by the specific sub-type and splice variant of the α_1_-subunit, the specific subunit-composition, phosphorylation status as well as the level of channel inactivation (for review, see [41]). 

The rationale for considering VGCCs as possible neuroprotective targets is based on the hypothesis that VGCCs respond to the persistent ischemia- or hypoxia-induced depolarization (reviewed in [42]). Based on their extremely fast inactivation kinetics [41,43], Cav3 channels are unlikely contributors to insult-induced Ca^2+^ load. The pivotal role of Cav2 channels in neurotransmitter release may contribute to an overall insult-induced state of neuronal hyperexcitability, however, their role in contributing to increases in intracellular Ca^2+^ following insult is questionable, given their localization at the active zone of synapses and their intermediate inactivation kinetics. In contrast, Cav1 (L-type calcium) channels exhibit slow voltage-dependent inactivation [41]. Furthermore, Cav1 channels have been shown to be physically and functionally linked to intracellular Ca^2+^ release channels such as RyR in neurons [44,45]. 

In *in vitro* systems, dihydropyridine blockers of Cav1 channels, such as nimodipine, israpidine, and nicardipine have shown neuroprotective effects by reducing the intracellular rise in Ca^2+^ following insult [46,47]. However, results from *in vivo* models have been highly variable: whereas some studies found neuroprotective effects of dihydropyridines [48,49,50,51,52,53,54,55,56,57], others failed to observe any effect on insult [for review, see 56,58]. The findings of the protective function of nimodipine in animal models of focal cerebral ischemia have been comprehensively reviewed [56]. 

Clinical trials have failed to show improved outcome after stroke with nimodipine treatment, as recently reviewed by Ginsberg [59]. It should be noted, however, that treatment was initiated at 24 or even 48 hrs after stroke in the majority of these earlier studies. Despite its unquestionable clinical relevance, this timeframe is well beyond the likely useful therapeutic window of nimodipine [59]. 

Despite these seemingly contradictory *in vivo* experiments and disappointing clinical trials, Cav1 channels remain a therapeutically-relevant target, as evidence emerges that other known neuroprotective compounds mediate their beneficial effects, at least in part, via Cav1 channels. Nicotine was shown to reduce glutamate excitotoxicity in mouse cortical cultures by lowering intracellular Ca^2+^ influx by ~40% through a pathway involving β_2_ nicotinic acetylcholinergic receptors, calcineurin and Cav1 channels [60]. Similarly, the hormone vitamin D exerted neuroprotective effects against excitotoxic insult in primary rat hippocampal cultures by reducing Cav1 expression, as determined by measuring L-type Ca^2+^ currents [61]. 

A novel broad class of Ca^2+^ channel blockers purified from the spider toxin of *Phoneutria nigriventer* has recently been shown to block ischemia-induced glutamate release, neuronal death, and loss of neurotransmission in hippocampal slice preparations subjected to oxygen deprivation and low glucose insult [62,63]. 

The complex regulation of intracellular Ca^2+^ homeostasis and the diverse effects of ischemic, excitotoxic or hypoxic insult thereupon may require more than a single molecular target for successful neuroprotective therapeutic intervention. Rational drug design efforts have recently provided promising results for neuroprotection: novel tacrine-dihydropyridine hybrids that inhibit both acetylcholinesterase and L-type Ca^2+^ channel-mediated rises in intracellular Ca^2+^ have been shown to exert neuroprotective effects in a cellular model for excitotoxic insult [64]. 

Despite disappointing results from early *in vivo* animal studies and clinical trials, reducing the activity of dihydropyridine-sensitive Ca^2+^ channels must continue to be considered in future rational drug design approaches for neuroprotection.

## 4. Ionotropic Neurotransmitter Receptors and Their Associated Proteins Controlling Intracellular Calcium Signaling as Targets for the Development of Neuroprotective Strategies

### 4.1. N-methyl-D-aspartic acid (NMDA) receptor activity and neuronal cell death

The role that NMDA receptors play in long-term potentiation, synaptic plasticity and excitotoxicity in the brain has been extensively studied and is reviewed in detail elsewhere [65,66,67]. In brief, Ca^2+^ influx through NMDA receptors and subsequent activation of a variety of signaling events ultimately leads to the expression of proteins involved in synaptic plasticity (reviewed in [67]). Overstimulation of NMDA receptors by glutamate, however, leads to excessive Ca^2+^ influx resulting in calpain activation and cell death [68]. In retinal ganglion cells (RGCs), glutamate treatment activates NMDA receptors resulting in delayed intracellular Ca^2+^ dysregulation and cell death while antagonists of NMDA receptors provide protection against toxicity [69]. Numerous basic science studies and clinical trials suggest that NMDA receptor antagonists, such as memantine, show some efficacy as a neuroprotectant in AD, especially if used in combination with other therapies (reviewed in [70,71]). Other novel potential therapeutic targets for neurodegenerative diseases include the modulation of intracellular Ca^2+^ channel activity and disruption of scaffolding proteins that couple glutamate receptor activity to Ca^2+^ release from intracellular stores.

### 4.2. Functional coupling of intracellular Ca^2+^ channels to ionotropic glutamate receptors through scaffolding proteins

Given the critical function of intracellular Ca^2+^ signaling and ICCs in a variety of cellular functions including protection from cellular insults, scaffolding proteins that structurally or functionally couple them to ionotropic glutamate receptors (iGluRs) are of great importance to the maintenance of normal cellular function. Targeting of a variety of excitatory ionotropic neurotransmitter receptors is achieved through scaffolding proteins such as PSD-95, Chapsyn-110, SAP97, PICK-1, gephyrin, GRIP, ABP and GIPC [72,73,74,75,76,77,78,79,80,81,82,83,84,85]. The importance of ionotropic neurotransmitter receptor targeting underscores the relevance of scaffolding proteins. The activity of ICCs is regulated in part by plasma membrane depolarization by AMPA receptors and subsequent Ca^2+^ influx generated from NMDA receptors [86,87,88,89] making the regulation of iGluRs important for ICC function and overall intracellular Ca^2+^ signaling. 

Actin, itself, can bind to and alter the function of IP_3_Rs [90] and actin depolymerization provides a means for ICC regulation [90,91,92,93]. Likewise, treatment of cultured hippocampal neurons with actin destabilizing agents reduces the number of GluR1 AMPA subunit and NR1 NMDA subunit clusters in the dendritic spines of pyramidal neurons [94]. The cytoskeleton also regulates NMDA receptor activity in neurons [95]. Given the importance of actin binding to IP_3_Rs, NMDA and AMPA receptor subunits, alteration of the actin cytoskeleton may also be a mechanism by which synaptic Ca^2+^ signals are regulated.

Several studies provide evidence that synaptic scaffolding proteins are critical for maintaining proper neuronal function and viability. Treatment of cultured mouse cortical neurons with F-actin destabilizing agents selectively disrupts NMDA receptor but not AMPA receptor clustering in dendritic spines [96]. Furthermore, disrupting dendritic NMDA receptor clusters with F-actin destabilizing agents reduces excitotoxic cell death in response to increased synaptic glutamate release caused by oxygen-glucose deprivation [96].

Using recombinant mutant NMDA receptor subunits, it was shown that NR2 subunits are required to effectively recruit NR1-1a splice variant subunits into dendritic spines [97]. This effect was partially dependent upon the PDZ domains in NR2 subunits. 

### 4.3. Ionotropic glutamate receptor function in neurodegenerative disease models

NMDA receptors are affected in models of AD. Treatment of cortical neurons with Aβ_1-42_ peptide increases endocytosis of NMDA receptor subunits (NR1 and NR2B) [98]. In addition, animals overexpressing Aβ_1-42_ exhibit a reduced surface NMDA receptor density [98]. Cultured neurons from mutant APP transgenic mice exhibit reduced PSD-95 expression and GluR1 AMPA receptor subunit surface expression [99]. PSD-95 knockout mice exhibit an increased number of silent synapses due to a reduced number of functional AMPA receptors [100]. NMDA receptor decay kinetics are also altered due to the possible increase in NR2B *versus* NR2A NMDA receptor subunit expression [100]. PSD95 targets nNOS to the NMDA receptor thereby coupling Ca^2+^ influx to nNOS activation [101]. Nitric oxide (NO) produced from neuronal nitric oxide synthase (nNOS) is a mediator of excitotoxicity and disruption of PSD95-nNOS interaction may provide protection from excitotoxicity [101]. Reduction of PSD-95 expression in cultured cortical neurons reduced NMDA receptor-mediated excitotoxicity by uncoupling NMDA receptors to nitric oxide synthase (NOS), a significant contributor to excitotoxic neuronal death [102].

Aβ dimer and trimer treatment of hippocampal tissue slices leads to a significant decrease in the number and density of dendritic spines in pyramidal neurons [103]. Furthermore, oligomeric Aβ treatment of slice cultures lead to a reduction in NMDA activity [103]. In addition, inhibition of NMDA receptors prevents Aβ-mediated dendritic spine loss [103]. Oligomeric Aβ treatment significantly reduced Ca^2+^ influx though NMDA receptors. In addition, the oligomeric Aβ -mediated reduction in spine density is dependent upon the phosphatase calcineurin and actin destabilization [103]. Overexpression of APP in rat organotypic hippocampal slice cultures results in a reduction in the number of dendritic spines [104]. Furthermore, Aβ reduces glutamatergic transmission through a mechanism similar to long-term depression (LTD) [104]. Hippocampal slices overexpressing APP exhibit reduced excitatory synaptic transmission perhaps providing a negative feedback mechanism for overexcitation and excitotoxicity [105].

Introduction of a GluR2 derived peptide containing a GRIP- and ABP-interacting PDZ domain into hippocampal slices increased AMPA-mediated excitatory postsynaptic current in pyramidal neurons in addition to reducing LTD [78] suggesting that GRIP and ABP reduce AMPA receptor reinsertion. Furthermore, PKC phosphorylation of AMPA receptors increases AMPA receptor insertion into the plasma membrane thereby preventing it from interacting with GRIP and ABP [78]. The presence of GluR2 AMPA receptor subunits leads to reduced ion conductance and subsequent excitotoxicity [106]. In a traumatic brain injury model, phosphorylation and subsequent internalization of GluR2 by the PICK1-PKCα-PSD-95-NMDA receptor complex results in excitotoxicity [106].

### 4.4. Glutamate receptor-ICC coupling as a therapeutic target in neurodegeneration

NMDA receptors and ICCs functionally interact through a calcium-induced Ca^2+^ release (CICR) mechanism mediated by RyR and also by regulation of IP_3_Rs by elevated cytosolic Ca^2+^ concentrations [86,87,88,89,107,108,109,110]. In addition, IP_3_R and RyR activity is regulated in part by posttranslational modifications, such as phosphorylation, triggered by upstream signaling events including Ca^2+^ influx into the cell [111,112,113,114,115,116,117,118,119,120,121,122,123,124,125]. 

Furthermore, iGluRs and ICCs interact through a complex network of scaffolding proteins in the postsynaptic density [126,127,128,129]. All of these interactions of iGluR with ICC are influenced by scaffolding proteins, which provide a structural assembly mediating the clustering of receptors and facilitating an increase in the proximity among receptor types. Clustering and targeting of iGluRs at synapses, for example, is more efficiently achieved through scaffolding proteins [73,74,80,81]. The trafficking and targeting of ionotropic neurotransmitter receptors to the plasma membrane and physiologically relevant sites such as synapses is critical for neuronal function especially in the context of synaptic plasticity and neuronal cell death. Disruption of iGluR targeting or surface expression of iGluRs likely has a significant effect on ICC activity since plasma membrane depolarization and Ca^2+^ influx will be reduced. 

## 5. G-protein Coupled Receptors (GPCR) Linked to the Control of Intracellular Calcium Signaling as Targets for the Development of Neuroprotective Strategies

### 5.1. G-protein coupled receptor signaling

G-protein coupled receptors (GPCR) are heptahelical plasma membrane proteins, which relay information on extracellular conditions to the cytosol. Despite the large variety of GPCRs, signaling is conveyed through a relatively small number of conserved G-protein signaling pathways. Upon receptor activation the Gα subunit is activated by binding of GTP and dissociation from the inhibitory Gβ/γ dimer [130]. G-protein signaling is sorted into four classes by the effects of the Gα subunit - Gα**_s_**, Gα**_i/o_**, Gαq, and Gα12 [131]. Gα**_s_** (stimulatory) and Gα**_i/o_** (inhibitory) modulate the production of cAMP by adenylyl cyclase. cAMP regulates cAMP-dependent protein kinase A (PKA) which affects downstream processes such as cAMP response element binding (CREB) protein activation, neuronal excitability, and gene transcription [130,132]. Phospholipase C (PLC) cleaves phosphatidylinositol bisphosphate (PIP2) to diacylglycerol and IP_3_ which then activate protein kinase C (PKC) and calcium release by IP_3_Rs, respectively. GPCR signaling mechanisms has been ably reviewed in [133,134] and Gαq signaling was recently reviewed in detail [135].

### 5.2. Cannabinoid receptors as targets for GPCR-mediated neuroprotection

The type 1 cannabinoid receptor (CB1) is expressed in the central nervous system (CNS) and has a strong anti-glutamatergic effect. Two endogenous ligands have been identified for CB1, anandamide [136] and 2-arachidonyl glycerol (2-AG) [137,138]. A peptide has recently been proposed as a third endogenous ligand [139]. CB1 receptors are classically thought to inhibit cAMP accumulation in a Gα**_i/o_** dependent manner [140,141], but numerous studies have found CB1 linked to calcium responses of interest to neuroprotection. The CB1 agonist WIN55,212-2 was found to inhibit presynaptic N-type and P/Q-Type voltage gated calcium channels (VGCC) [142,143,144] and inhibited the high-voltage-activated Ca^2+^ current in rat retinal ganglion cells [145]. WIN55,212-2 also generated a Gαq response though only at relatively high concentrations of 5 µM [146]. CB1 receptors have been found at high densities on the presynaptic terminal of glutamatergic synapses [142]. Agonism of CB1 reduced NMDA excitotoxicity [147,148,149,150,151] and inhibited synaptic glutamate release [152]. A PKA-dependent mechanism prophylactic of NMDA excitotoxicity was described by Kim *et al.* [153]. Zhuang *et al.*, correlate this protective effect to CB1-mediated inhibition of PKA which leads to dephosphorylation of the ryanodine receptor and decreased intracellular calcium release [154]. Promiscuity of the CB1 receptor makes it difficult to attribute its neuroprotective effect to a single action. Though the majority of the data suggests the NMDA receptor as the likely effector, a mechanism has yet to be determined

### 5.3. Retinal neuroprotection through the α2 adrenergic receptor

Glaucoma is characterized by increased intraocular pressure (IOP) and leads to retinal cell death and blindness [155,156]. Decreased blood flow of the glaucomatous eye indicates the neuronal damage may be similar to glutamatergic ischemic damage observed elsewhere in the CNS [155]. The α2 adrenergic receptor has been found to decrease glutamate toxicity in the retina [157,158]. NMDA receptors have been detected in the healthy retina [159,160] and toxic concentrations of glutamate were found in the vitreous humor of the glaucomatous eye [161,162,163,164]. Treatment with NMDA antagonists is not a viable solution due to side effects [165,166]. The NMDA partial agonist memantine was neuroprotective *in vitro* and certain rodent studies [167,168,169,170] but when tested in macaque monkeys memantine only reduced the rate of retinal ganglion cell (RGC) loss and showed no effect on total RGC survival [171,172]. Agonists of the α2 adrenergic receptor have shown promise in preventing cell death [173]. The α2 receptor is expressed in the retina though at a lower level in the outer nuclear layer (ONL) than in the inner layer (INL) [174]. The α2 agonist brimonidine has shown clinical efficacy in reducing IOP [156,175], however, proof of a neuroprotective activity and mechanism is lacking in humans [156]. Pretreatment with brimonidine prevented an increase of the vitreous glutamate concentration and blocked deterioration of the electroretinogram (ERG) b-wave in a rat model of transient retinal ischemia [157]. Brimonidine treatment after retinal damage failed to prevent RGC death [173]. Pretreatment of rat RGCs with the α2 agonists brimonidine or medetomidine inhibited calcium influx through the NMDA receptor and L-type VGCC in the INL but not in the ONL [176]. The observed decrease of calcium influx seems to have been α2-mediated but it is not clear if the effect was due to a Gα**_i/o_** mechanism or α2 receptor activation of the inward rectifying K^+ ^channel [177]. A follow-up study found that brimonidine neuroprotection against NMDA toxicity required a decrease of the intracellular cAMP concentrations by either inhibition of cAMP-specific phosphodiesterase (PDE) or inhibition of adenylyl cyclase [178]. Alternatively, the Gβ/γ subunits of the inhibitory G-protein trimer have been found to inhibit VGCCs [179] though that does not seem to be the case in the INL or ONL [176]. Further clarification of the mechanism of action is required but α2 agonists are potentially powerful tools for the treatment of neurodegenerative disorders. 

## 6. Presenilins as Disease-Relevant Proteins Involved in the Control of Intracellular Calcium Signaling as Targets for the Development of Neuroprotective Strategies

Presenilins (PS) are ubiquitously expressed in most tissues [180,181,182,183,184,185,186] and have been implicated as a causal factor in AD disease progression [1,4,8,9,10,11,12,13,14,25,32,180,183,187,188,189,190,191]. PS is a 9-transmembrane aspartyl protease expressed on the ER membrane with two variants (PS1 and PS2) [192,193,194,195]. While on the ER, PS is cleaved between the 6^th^ and 7^th^ transmembrane domains [25,188,193,195]. Both fragments re-associate and are trafficked to the plasma membrane where they form the proteolytic core of the γ-secretase complex which cleaves APP including production of Aβ [25,180,187,189,191,195,196]. The effects of PS mutations on Ca^2+^ regulation are profound though mixed [29,31,97,98,99,100,101,102,103,104,105,106,107,108,109,110,111,112,113,114,115,116,117,118,119,120,121,122,123,124,125,126,127,128,129,130,131,132,133,134,135,136,137,138,139,140,141,142,143,144,145,146,147,148,149,150,151,152,153,154,155,156,157,158,159,160,161,162,163,164,165,166,167,168,169,170,171,172,173,174,175,176,177,178,179,180,181,182,183,184,185,186,187,188,189,190,191,192,193,194,195,196,197,198,199,200,201,202,203,204,205]. The genetics of AD [206], PS signaling [180] and aspects of calcium signaling in AD [25,238] were reviewed recently.

Toxic concentrations of Ca^2+^ in the cytosol have been implicated in neurodegenerative disorders like AD [25,191,202,207,208]. Ischemic conditions are also characterized by increased cytosolic calcium concentrations [200,209,210,211,212]. Hypoxia increased PS2 mRNA 10-fold [213] and PS2 expression levels returned to normal within 48 hours of reperfusion [213], potentially indicating increased Ca^2+^ release during ischemia as a causative mechanism [200,211]. 

Rybalchenko *et al.* [27] and Hayrapetyan *et al.* [28] have recently described a novel mechanism where the N-terminus of PS (PS-NTF) binds to the RyR to increase the mean calcium current and open probability [27,28]. Increased RyR open probability resembles an ER calcium leak due to the low level, partial activation of a significant share of RyR while the cell is at rest [27,28]. Binding of PS-NTF to RyR could stabilize the open state of the receptor or decrease the energy required for the conformational shifts between closed and open states. PS1-NTF and PS2-NTF provoked very similar responses when bound to the RyR, both fragments increased the amount of time the receptor spent in the open state but neither fragment induced channel gating at sub-threshold calcium concentrations (100 nM Ca^2+^) [27,28]. On the other hand, PS2-NTF blocked desensitization of RyR by inhibitory concentrations of Ca^2+^ (>1 mM) [27,28] but treatment with PS1-NTF had no effect on calcium inhibition of RyR gating [27,28]. These results indicate that PS1 and PS2 are positive allosteric modulators of RyR that increase the potency of the receptor’s endogenous agonist, Ca^2+^.

An interaction between PS and RyR had been described in other parts of the literature though a specific functional molecular interaction was novel to Rybalchenko *et al.* [27] and Hayrapetyan *et al.* [28]. Both NTF and CTF of PS1 were precipitated with RyR2 in PC12 cells [26]. The cleaved CTF of PS2 has been detected bound to RyR [30]. PC12 cells stably transfected with PS1 up-regulate the expression of RyR3 [26]. The same study also found RyR3 up-regulation in a mouse knock-in model of common FAD PS1 mutations [26]. PS has also been reported to act as an ER calcium leak channel [201,214]. In contrast, another study using a different model system strongly contradicts the conclusions of Tu and colleagues [32]. Sorcin stabilizes RyRs which increases calcium release [216]. The CTF of proteolytically cleaved PS2 binds to sorcin in a calcium dependent manner [215]. Though no direct link between the PS2-sorcin complex and RyR has been described, SH-SY5Y cells transfected to overexpress PS2 did show increased cytosolic calcium release [215], hypothesized due to a PS2-sorcin-RyR interaction [215].

Cheung *et al.* (2008) described an analogous interaction between PS and a second class of ER calcium release channels, the IP_3_R [32]. Cheung *et al.* (2008) reported a PS-IP_3_R interaction that increased receptor open probability and calcium release [32]. This paper’s conclusion contradicts data indicating increased IP**_3_** hydrolysis by phospholipase C-β (PLCβ) in human neuroblastoma SH-SY5Y cells transfected with mutant PS1 containing an exon 9 deletion (PS1ΔE9) [188]. PS1ΔE9 is a splice variant that is not proteolytically cleaved and therefore not trafficked to the plasma membrane [188]. PS1ΔE9 transfected cells stimulated with the muscarinic agonist carbachol displayed increased calcium release but wild type (WT) cells and PS1 transfected cells showed no significant response [188]. Interestingly, human PS2 but not PS1 increased IP_3_R Ca^2+^ signaling in a *Xenopus* oocyte system [31]. 

To date, studies indicate a causal relationship between ER Ca^2+^ release and PS. Much more work is required to fully define the role of PS in ER Ca^2+^ release but the current data indicate therapies could be developed to blunt excessive Ca^2+^ release found in neurodegenerative conditions like AD and ischemia.

## 7. New Imaging Technologies for Studying the Role of Intracellular Calcium Signaling

Despite the rapidly evolving knowledge on the role of aberrations in Ca^2+^ signaling in both acute and chronic neurodegenerative disease, progress in the development of neuroprotective approaches targeting the control of the intracellular Ca^2+^ concentration have been limited, largely due to the lack of knowledge about the spatio-temporal properties of Ca^2+^ signaling in different subcellular domains. However, in recent years a number of novel imaging technologies have become available that will help the accurate measuring the intracellular Ca^2+^ concentration in different sub-compartments and domains of the cell. In the following two subsections advances in the design of novel genetically-encoded Ca^2+^ sensors and progress in new superresolution microscopy will be reviewed and their potential use for the development of novel neuroprotective strategies be discussed. 

### 7.1. Small molecule and genetically encoded Ca^2+^ sensors

Several new technologies are becoming available for the imaging and quantification of cellular processes by microscopic observation. Some research groups, such as the Tsien laboratory have been working to improve the kinetics, stability, and range of fluorescent sensors [217]; while others, including the Lippincott-Schwartz group, have been creating technologies that expanded the spatial and temporal resolution of microscopic observation and recording in live cells [218]. Small molecule and genetically encoded Ca^2+^ sensors are characterized by both strengths and weaknesses associated with these different approaches to Ca^2+^ imaging and quantification. In addition, current superresolution microscopy technologies expand the spatial resolution of light microscopy resulting in improved strategies for measuring intracellular Ca^2+^ signaling.

The intensity, quantum efficiency, stability, membrane permeability, toxicity, and kinetic parameters determine the overall experimental usefulness of Ca^2+^ sensor dyes. No ideal strategy exists for intra-cellular Ca^2+ ^imaging resulting in the need for compromises in experimental design.

The small molecule sensors of intracellular Ca^2+^ levels are epitomized by the Fura family of calcium sensitive binding dyes. Ratiometric imaging of the Fura2 dye, developed by the Tsien group in 1985, is considered to be the best method for quantification of Ca^2+^ concentration in living cells [217,219]. Typically cells are loaded using the plasma membrane permeable acetoxymethyl ester of Fura2 (Fura2-AM). The acetoxymethyl ester moiety is hydrolyzed and cleaved from the Fura2 molecule by the action of endogenous esterases leaving the Fura2 molecule to interact with any free Ca^2+^ ions in the intracellular milieu. The Fura2 molecule acts as Ca^2+^ chelator, on binding to free Ca^2+ ^ions, Fura2 changes its peak absorbance wavelength from ~350 nm to~ 340 nm with a concomitant change, a decrease in absorbance at~380 nm, while its emission maxima remains at ~510 nm. After calibration of Fura2 fluorescent intensities with known Ca^2+^ concentrations, the intracellular concentrations of Ca^2+^ can be calculated based on the ratio of changes in fluorescent intensities at 510 nm with excitation at 340 nm and 380 nm in living cells. Fura2 has a Ca^2+^ binding affinity near physiologic levels and a high selectivity for Ca^2+^ ions [217,219]. The advantages of these small molecule indicators of Ca^2+^ includes high selectivity, fast kinetics, and the ability to quantify Ca^2+^ concentrations using ratiometric imaging. Ratiometric imaging techniques reduce quantification errors caused by indicator concentration, excitation intensity, and detector efficiency. However, ratiometric image analysis is subject to errors [220], resulting in the need for specific controls in the experimental design. 

Recently, Tsien and others have been focused on producing Genetically Encoded Ca^2+^ Indicators (GECIs). Sensors using Förster Resonance Energy Transfer (FRET) pairs or Bimolecular Fluorescence Complementation (BiMC) can be used to quantify Ca^2+^ levels using ratiometric techniques. However, the fusion of Fluorescent Proteins (FP’s) to calcium sensitive proteins is not a trivial task. Fusion of FPs can change the behavior of the sensor, change the protein folding pattern, or cause autophagocytosis of the sensor and in addition consideration of Heisenberg’s uncertainty principle of quantum theory [221] is needed as the mere presence of either small molecule sensors or genetically encoded sensors, or even the illumination used to probe the sensors may perturb the cellular mechanism that is being measured. 

Tsien’s group has produced directed mutations of Green Fluorescent Protein (GFP) from *Aequorea victoria* and DsRed from *Discosoma sp.* to form a wide spectrum of genetically encoded fluorescent protein variants. These new genetically encoded fluorescent proteins can be used for multiple spectral and temporal labeling of target proteins and events. These fluorescent proteins can be fused to calcium chelators containing the C2 domain or the EF hand domain to create genetically encoded Ca^2+^ sensors, whose construction, coordination, and geometry has been reviewed recently [222]. These constructs can be generated as single fluorophores such as Camgaroo and Peircam, or as FRET based dual fluorophores. 

GECIs have expanded the experimental capabilities including targeting the sensor to specific organelles or cellular sites, the generation of transgenic animals, conditional expression, and photo-activation of the indicators. At the same time, the genetically encoded sensors do not share the fast kinetics of the small molecule sensors. The slow kinetics and the lower signal to noise ratio prevents detection of fast or weak changes in calcium concentration. However, GECIs continue to be improved. Recently, Tian *et al.* used structure guided engineering to produce GCaMP3, which has a 3 fold increase in fluorescence, a 3 fold increase in dynamic range, and a 1.3 fold higher affinity for calcium than its parental GCaMP2 sensor [223]. The future of Ca^2+^ analysis *in vivo* will likely be dominated by improvements of sensor signal to noise ratios and of binding affinities. 

### 7.2. Superresolution microscopy

Advances in light microscopy have led to the breaking of the diffraction limit of resolution. These new methods have been referred to as superresolution microscopy or nanoscopy [224,225]. Until recently light microscopy was limited to about 180-220 nm resolution laterally and 500–800 nm axially [226,227]. However several new techniques have been implemented which break the diffraction limited resolution barrier, which have also been reviewed recently [227]. 

Impressive sub-diffraction limit resolution of point fluorescence sources has been achieved using these methods [227,228,229,230,231,232,233,234,235,236]; however, superresolution imaging is not a trivial exercise. Superresolution techniques usually require extremely precise alignment of multiple laser sources, and optical components in addition to requiring substantial computational resources [227]. Gains in axial and lateral resolution may be offset by losses in temporal resolution due to the limitations of computational resources. Optical techniques such as Stimulated Emission Depletion (STED) also face wavelength limitations [227,228,229]. 

The combination of new dye technologies with dramatically improved binding affinities and faster kinetics with robust superresolution techniques will be instrumental in measuring the intracellular Ca^2+^ concentration and study the effects of novel neuroprotective compounds. 

## 8. Conclusions

Disease-mediated changes in the regulation of intracellular calcium signaling are characteristic for acute and chronic degenerative diseases of the nervous system as evidenced by studies at the cellular and system level. While the homeostasis of the intracellular calcium concentration is the ultimate readout, numerous molecular and cellular components contribute to the overall change and at the same time represent qualified targets for the design of neuroprotective strategies. The capacity to both identify such targets as well as to reproducibly measure effects on the regulation of intracellular calcium signaling determine critically the success of neuroprotection.
